# The emerging role of nutrition in Parkinson's disease

**DOI:** 10.3389/fnagi.2014.00036

**Published:** 2014-03-07

**Authors:** Stacey E. Seidl, Jose A. Santiago, Hope Bilyk, Judith A. Potashkin

**Affiliations:** ^1^The Cellular and Molecular Pharmacology Department, The Chicago Medical School, Rosalind Franklin University of Medicine and ScienceNorth Chicago, IL, USA; ^2^The Nutrition Department, The College of Health Professions, Rosalind Franklin University of Medicine and ScienceNorth Chicago, IL, USA

**Keywords:** Parkinson's disease, nutrition, neurodegeneration, neuroprotection, antioxidants

## Abstract

Parkinson's disease (PD) is the second most prevalent neurodegenerative disease in ageing individuals. It is now clear that genetic susceptibility and environmental factors play a role in disease etiology and progression. Because environmental factors are involved with the majority of the cases of PD, it is important to understand the role nutrition plays in both neuroprotection and neurodegeneration. Recent epidemiological studies have revealed the promise of some nutrients in reducing the risk of PD. In contrast, other nutrients may be involved with the etiology of neurodegeneration or exacerbate disease progression. This review summarizes the studies that have addressed these issues and describes in detail the nutrients and their putative mechanisms of action in PD.

## Introduction

Parkinson's Disease is a neurodegenerative disease that usually develops late in life and is characterized by the loss of dopaminergic neurons in the substantia nigra pars compacta (SNpc). Most cases of Parkinson's disease (PD) are idiopathic since their cause is unknown. Genetic susceptibility and environmental factors (Warner and Schapira, 2003) that mediate mitochondrial dysfunction, inflammation, abrogation of the autosomal-lysomal autophagy system (Beal, 2003), and endoplasmic reticulum stress (Ryu et al., 2002) play a role in disease development.

A growing body of evidence suggests that nutrition may play an important role in PD. Epidemiological and biochemical studies have recently identified promising components in certain food groups that may elicit neuroprotection in PD (Searles Nielsen et al., 2013; Shaltiel-Karyo et al., 2013). However, inclusion or exclusion of other food groups may trigger or exacerbate neurodegeneration. In this review, we focus on the role nutrition plays in promoting or slowing PD.

## Nutrients that may be associated with an increased risk or progression of PD

### Dairy products

Dairy product consumption and drinking milk may increase one's risk of PD independently of calcium intake (Hellenbrand et al., 1996b; Chen et al., 2002; Park et al., 2005; Kyrozis et al., 2013), particularly in men (Chen et al., 2007a). Nonetheless, a positive association between milk consumption and PD risk was also observed in women in one study (Saaksjarvi et al., 2013). Preliminary research shows that individuals who consume large amounts of dairy products may often have low serum uric acid levels (Choi et al., 2005a). Serum urate and uric acid is inversely correlated with the risk of PD and disease duration (Weisskopf et al., 2007; Schlesinger and Schlesinger, 2008; Andreadou et al., 2009; Shen et al., 2013). The neuroprotective effects of serum urate may be limited to men (Gao et al., 2008; Shen et al., 2013) since the same is not observed in women (O'Reilly et al., 2010). In addition, the possible presence of dopaminergic neurotoxins, including pesticides and polychlorinated biphenyls in dairy products may increase the risk of PD (Chen et al., 2002). Accordingly, postmortem studies show higher levels of organochlorines, including dieldrin, an organochlorine pesticide, and polychlorinated biphenyls in the brains of PD patients compared to non-neurological controls (Fleming et al., 1994; Corrigan et al., 1998). Yet, the presence of dopaminergic neurotoxins may not be the only component responsible for the relationship between dairy products and PD. In fact, a strong positive association with the consumption of milk, but not cheese or yoghurt has been reported (Kyrozis et al., 2013). Therefore, other constituents in milk may be detrimental with regards to PD and additional studies are needed in order to identify them. The association between dairy products and PD should be interpreted with caution, however, as other studies have found conflicting results (Miyake et al., 2011c).

## Nutrients that may be associated with a decreased risk or progression of PD

### Phytochemicals

The health benefits associated with the intake of phytochemicals present in fruits and vegetables leads to decreased functional decline associated with aging and may slow the progression of PD (Liu, 2003). Epidemiological studies found that high intake of fruits, vegetables and fish was inversely associated with PD risk (Gao et al., 2007; Okubo et al., 2012). Dietary patterns, characteristic of a Mediterranean diet, are emerging as a potential neuroprotective alternative for PD (Alcalay et al., 2012).

Most fruits and vegetables are rich sources of antioxidants, including vitamins A, B (riboflavin), C, and E, which are present in low levels in some PD patients. Numerous studies have reported a decrease in peroxidase (Ambani et al., 1975), glutathion-peroxidase activities (Kish et al., 1985), and glutathione (Riederer et al., 1989) in the SN of PD patients postmortem; suggesting metabolic failure in antioxidant mechanisms and chemical processes can lead to lipid peroxidation and parkinsonian characteristics (Uttara et al., 2009).

Although the antioxidant capacity of some fruits and vegetables is evidenced in numerous studies, a recent investigation raised caution about the antioxidant properties of pomegranate. Contrary to the previously reported neuroprotective effects observed in Alzheimer's Disease (Hartman et al., 2006), pomegranate juice exacerbated oxidative stress and neurodegeneration in a rotenone model of PD (Tapias et al., 2013). However, the authors suggest that oxidative stress in a rotenone model may be substantially overwhelming and promegranate may act as a pro-oxidant.

Epidemiological studies have found a decrease in PD risk in individuals who consume foods containing carotenoids and β-carotene (Miyake et al., 2011a). Carotenoids possess antioxidant properties; they act as a reducing agent by protecting lipids through oxidation interference and free radical entrapment (Paiva and Russell, 1999). In mice, pretreatment with β-carotene partially protected against MPTP-induced neurotoxicity (Perry et al., 1985; Yong et al., 1986), but not in primates (Perry et al., 1987). Lycopene, another carotenoid compound, reduces oxidative stress and cognitive decline in a rotenone-induced rodent model of PD (Kaur et al., 2011). One should be cautious however about applying conclusions from animal models about the benefits of carotenoids to humans, since most animals do not absorb or metabolize carotenoids in a similar manner (Paiva and Russell, 1999).

Riboflavin is an integral component of the coenzymes flavin adenine dinucleotide and flavin mononucleotide. Flavin coenzymes participate in oxidation–reduction reactions where they are a major source of energy and are critical for carbohydrate, fat and protein metabolism (Massey, 2000). It has been suggested that riboflavin may be involved in glutathione depletion, cumulative mitochondrial DNA mutations, disturbed mitochondrial protein complexes, and abnormal iron metabolism (Coimbra and Junqueira, 2003). Despite these characteristics, some studies found that riboflavin is not associated with the risk of PD (Abbott et al., 2003; Murakami et al., 2010b), whereas another study observed improved motor skills in PD patients with daily supplementation of riboflavin for 6 months and elimination of red meat (Coimbra and Junqueira, 2003). However, several limitations of this study including omission of a placebo control group and the investigators not being blinded have lead others to question these findings (Ferraz et al., 2004). Another important consideration is that lower protein consumption may affect the absorption of levodopa (Pare et al., 1992; Crevoisier et al., 2003). Therefore, the apparent benefit in motor skills could have resulted from a better absorption of levodopa as opposed to riboflavin supplementation (Ferraz et al., 2004). In addition, intake of other related B vitamins including folate, vitamin B6 and B12 are not associated with a risk of PD (Chen et al., 2004). However, low intake of vitamin B6 is associated with an increased risk of PD (Murakami et al., 2010b). Larger placebo controlled blinded studies done over a longer period of time would be beneficial for determining if riboflavin or other related B vitamins are useful supplements for PD patients.

Recently, dietary intake of nicotine-containing vegetables from edible *Solanaceae* including tomatoes, potatoes, and peppers, was associated with a reduced risk of PD in men and woman who had never smoked cigarettes or tobacco (Searles Nielsen et al., 2013). It remains unclear as to whether the observed protective effect was due to the nicotine content or other components of this group of vegetables. Cruciferous vegetables such as cauliflower, cabbage, and broccoli, are another group of vegetables rich in antioxidants with neuroprotective capacity. For example, sulforaphane and erucin, are potent naturally occurring isothiocyanates found in cruciferous vegetables with antioxidant properties. Treatment with sulforaphane ameloriated motor deficits and protected dopaminergic neurons in a 6-OHDA mouse model of PD (Morroni et al., 2013). Similarly, erucin provided neuroprotective effects by preventing oxidative damage induced by 6-OHDA in an *in vitro* model (Tarozzi et al., 2012). Both, sulforaphane and erucin appear to be promising neuroprotective agents in chronic neurodegenerative diseases (Tarozzi et al., 2013). Taken together, these findings highlight the effects of some vegetables, fruits and constituents they contain as having neuroprotective potential.

### Omega-3 (DHA)

Omega-3 polyunsaturated fatty acids (PUFAs) appear to be neuroprotective for several neurodegenerative diseases (Bousquet et al., 2011a). There have been no studies in PD patients that address whether omega-3s are neuroprotective, however, one study showed that supplementation with omega-3 PUFA reduced depression in PD patients (Da Silva et al., 2008). Current research focuses specifically on the omega-3 fatty acid docosahexaenoic acid (DHA). DHA is an essential factor in brain growth and development (Horrocks and Yeo, 1999) and has anti-inflammatory potential due to its ability to inhibit cyclooxygenase-2 (Massaro et al., 2006). DHA protects neurons against cytotoxicity, inhibition of nitrogen oxide (NO) production, and calcium (Ca^2+^) influx. DHA also increases the activities of antioxidant enzymes glutathione peroxidase and glutathione reductase (Wang et al., 2003). Furthermore, DHA supplementation reduced apoptosis in dopaminergic cells (Ozsoy et al., 2011) and replaced omega-6-PUFAs in the brains of mice post-MPTP treatment (Bousquet et al., 2008). Short-term administration of DHA reduced levodopa-induced dyskinesias in parkinsonian primates by up to 40% (Samadi et al., 2006). Long-term administration of uridine and DHA increased the amount of neural phosphatides in synaptic membranes (Wurtman et al., 2006) and dendritic spines in rodents (Sakamoto et al., 2007). In addition, a reduction in parkinsonian behaviors and elevated dopamine (DA) levels in 6-OHDA rodents was observed after treatment with these supplements (Cansev et al., 2008). Further research on DHA intake in PD patients is needed to assess whether it is beneficial in slowing disease progression.

The protective effects of DHA are mediated by a metabolic derivative known as neuroprotectin D1 (NPD1) (Bazan, 2009; Serhan and Petasis, 2011). NPD1 protects neurons against oxidative stress, inflammation, the disruption of the cytoskeleton, and from the activation of apoptotic signaling pathways. DHA may protect the brain by increasing glutathione reductase activity that results in decreased accumulation of oxidized proteins (Calon et al., 2004; Wu et al., 2004), lipid peroxide and reactive oxygen species (ROS) (Hashimoto et al., 2005). DHA also inactivates caspase activation signaling pathways (Calon et al., 2005), inhibits hyperphosphorylation of tau (Green et al., 2007) and regulates the PI3K/Akt cascade (Akbar and Kim, 2002). Other potential mechanisms of action of DHA include regulation of inflammation, transcription, and cell membrane properties (De Urquiza et al., 2000; Salem et al., 2000; Jump, 2002).

The precursor to DHA, eicosapentaenoic acid (EPA) is neuroprotective in experimental models of PD (Song et al., 2009; Meng et al., 2010; Taepavarapruk and Song, 2010; Luchtman et al., 2012). In *in vitro* models of PD, EPA attenuated an MPP^+^-induced reduction in cell viability and suppressed pro-inflammatory cytokines (Luchtman et al., 2013). A diet rich in EPA diminished hypokinesia induced by MPTP in mice and ameliorated procedural memory deficit (Luchtman et al., 2013).

Because DHA and EPA provide neuroprotection in animal models, more research is warranted to determine if they are beneficial for PD patients. This could be accomplished by following a large group of individuals at risk for PD, some of who are randomly chosen to receive a supplement and other who receive a placebo. The participants could be followed over several years to determine if they develop PD. Alternatively, a large intervention study testing supplements in patients at various stages of PD might reveal whether motor and/or cognitive symptoms are reduced.

### Soy (genistein)

The primary soybean isoflavone genistein is a source of protein that appears to be neuroprotective in ovariectomized rats following 6-OHDA injection, thus suggesting it may be useful for the prevention of PD in post-menopausal women (Kyuhou, 2008). In PD, genistein treatment resulted in dopaminergic neuron protection from lipopolysaccharide (LPS)-induced injury via inhibition of microglia activation (Wang et al., 2005). Genistein pretreatment improved spatial learning and memory in parkinsonian rats (Sarkaki et al., 2009) and restored tyrosine hydroxylase (TH), dopamine transporter (DAT) and Bcl-2 mRNA expression in the midbrain of MPTP-treated animals (Liu et al., 2008). Restored levels of DA and its metabolites, dihydroxyphenylacetic acid, and homovanillic acid, in the striatum were also observed after genistein administration. Additionally, genistein attenuated rotational behavior, protected SNpc neurons (Baluchnejadmojarad et al., 2009), and preserved motor function (Kyuhou, 2008) from 6-OHDA toxicities. Genistein's neuroprotective actions may regulate mitochondria-dependent apoptosis pathways and suppress ROS-induced NF-κ B activation (Qian et al., 2012). These studies suggest that it may be worthwhile to test the neuroprotective benefits of genistein in a clinical trial.

### Caffeine

Caffeine is one of the most widely consumed substances. The health promoting benefits of caffeinated beverages is supported by numerous epidemiological studies (Prakash and Tan, 2011; Tanaka et al., 2011). An inverse association between PD and coffee, and caffeine from non-coffee sources, has been reported (Hellenbrand et al., 1997; Fall et al., 1999; Ascherio et al., 2001). In general, animal studies also indicate that caffeine is neuroprotective. The administration of caffeine to maneb- and paraquat-treated rodents reduced the number of degenerating dopaminergic neurons, microglial cells and nitrite content, while normalizing expression of IL-1β, p38 MAPK, NF-kB, and TK (Kachroo et al., 2010; Yadav et al., 2012). Acute and chronic administration of caffeine also reduced the effect of MPTP (Chen et al., 2001) and 6-OHDA treatment on striatal DA loss (Joghataie et al., 2004) and motor dysfunctions (Joghataie et al., 2004; Aguiar et al., 2006) in rats. Caffeine treatment partially restores DA metabolites in rats following 6-OHDA lesions (Aguiar et al., 2006), and provides neuroprotection in MPTP models of PD (Xu et al., 2010), thus extending its beneficial effects. It is important to note that a caffeine tolerance does not develop with long-term exposure in mice (Xu et al., 2002) and neuroprotection is still apparent with caffeine intake after the onset of neurodegeneration in rats (Sonsalla et al., 2012).

Genetic and pharmacological data from rodent studies indicate that caffeine reduces dopaminergic toxicity and slows disease progression through antagonism of adenosine A_2A_ receptors (Morelli et al., 2010; Prediger, 2010; Xiao et al., 2011; Sonsalla et al., 2012). Inhibition of glutamate neurotransmission using A_2A_ receptor antagonists, may relieve motor symptoms and provide neuroprotection in models of late-stage PD (reviewed in Popoli et al., 2004; Chen et al., 2007b). However, methylxanthine derivatives containing properties of monoamine oxidase B (MAO-B) inhibition, like 8-(3-chlorostyryl) caffeine, may cause oxidative stress via dysfunctional vesicular monoamine transporter 2 (VMAT2) and DA storage mechanisms early in PD (Golembiowska and Dziubina, 2012). Currently, clinical studies are underway to evaluate several A_2A_ receptor antagonists for symptomatic relief and slowing of disease progression (reviewed in Hickey and Stacy, 2011). Caffeine has also shown cytoprotective effects through activation of the PI3K/Akt signaling pathway in SH-SY5Y cells (Nakaso et al., 2008). Therefore, caffeine's ability to down-regulate NO production, neuroinflammation, and microglial activation through these pathways may contribute to neuroprotection (Yadav et al., 2012). It is not fully established, however, that caffeine's neuroprotective role is the sole reason for reduced risk of PD. Nor is it known whether the association is causal rather than reverse causation; the protective effect of caffeine could also reflect an effect of symptoms of PD on caffeine consumption.

Estrogen has significant effects on caffeine's neuroprotective capabilities. Epidemiological studies have consistently demonstrated a greater improvement in male than female Parkinson's patients (Ascherio et al., 2001; Costa et al., 2010). Interestingly, post-menopausal women who are not taking hormone-replacement therapy receive the same neuroprotective benefits as men (Ascherio et al., 2001). However, high caffeine consumption was associated with an increased risk of PD among women using hormones (Ascherio et al., 2003). More recently, findings from a larger prospective study are consistent with a neuroprotective effect of caffeine intake in men and an attenuated effect in women due to hormone replacement therapy (Palacios et al., 2012a). With regards to animal models, estrogen and caffeine co-administration in MPTP-treated mice, prevented neuroprotection in males and females (Xu et al., 2006). Together these studies suggest that the beneficial effects of caffeine may be limited to men and post-menopausal women not receiving hormone-replacement therapy. However, an open-label study examining caffeine's symptomatic effects and tolerability in patients demonstrated improved non-motor aspects of PD with no gender differences (Altman et al., 2011). Currently adenosine A_2A_ antagonists and caffeine are in phase II and III clinical trials for the symptomatic treatment of PD (Barkhoudarian and Schwarzschild, 2011).

### Tea

Several epidemiological studies have addressed the influence of drinking tea (Camellia sinensis) on the risk of PD. A case-control study of Chinese PD patients showed that regular tea drinking protects against PD (Chan et al., 1998). Another study complimented the Chinese PD study showing a reduced risk for PD with tea consumption (two cups/day) (Checkoway et al., 2002). Similarly, a large prospective study showed a reduced risk of incident PD in subjects who habitually drank three or more cups of tea per day (Hu et al., 2007). A retrospective study associated drinking of more than three cups of tea per day with a delayed onset of motor symptoms in Israeli PD patients (Kandinov et al., 2009). Unfortunately, no distinction between green and black tea was made in these studies.

Several reports have revealed that both black and green tea exert neuroprotective effects in PD animal models (Bastianetto et al., 2006; Chaturvedi et al., 2006). Polyphenols in green and black tea extracts provide highly potent antioxidant-radical scavenging activities in brain mitochondrial membrane fractions (Zhao, 2009). In addition, polyphenols in tea reduce occurrence of disease and provide neuroprotection in cell culture and animal models (Nie et al., 2002; Pan et al., 2003b; Guo et al., 2007). In black tea, the polyphenol theaflavin (TF) possess a wide variety of pharmacological properties including antioxidative, antiapoptotic, and anti-inflammatory effects (Aneja et al., 2004; Gosslau et al., 2011). TF-mediated neuroprotection against MPTP-induced dopaminergic neurodegeneneration in rodents was evidenced by increased expression of nigral TH, DAT and reduced expression of apoptotic markers (Anandhan et al., 2012).

Similarly, the polyphenol (–)-epigallocatechin-3-gallate (EGCG) in green tea shows promise in neuroprotection, but one study showed that green tea drinking was unrelated to the risk of PD (Tan et al., 2008). EGCG inhibits nitric oxide and tumor necrosis factor-α secretion from LPS-activated microglia in dopaminergic mesencephalic cells (Li et al., 2004). Given that microglia play a key role in the generation of free radicals and inflammatory factors in the brain, EGCG was classified as neuroprotective *in vivo* (Li et al., 2004). Additionally, EGCG improved cell viability and attenuated MPP-induced intracellular ROS formation via the SIRT1/PGC-1α signaling pathway in MPP induced PC12 cells (Ye et al., 2012). EGCG reduced neuronal cell death and induced nitric oxide synthase (NOS) expression in an MPTP mouse model of PD, thus providing further evidence for its neuroprotection via NO reduction (Kim et al., 2010). Oral pretreatment with EGCG prevented dopaminergic neuron loss in MPTP-treated mice (Levites et al., 2001). In contrast, another study found subtle symptomatic relief but no neuroprotection with similar dose of EGCG in rats following a 6-OHDA lesion (Leaver et al., 2009). The differences between the results from these two studies may reflect the different mechanisms by which MPTP and 6-OHDA exert their neurotoxic effects. Also, the poor bioavailability of oral EGCG in rats may explain why similar doses led to different results in animal models (Kim et al., 2000).

Computational molecular modeling has shown that EGCG is a potent, non-competitive inhibitor that invokes various cellular neuroprotection/neurorescue mechanisms (Zhu et al., 2008). EGCG's mechanisms of action include iron-chelation, scavenging of oxygen and nitrogen radical species, activation of protein kinase C (PKC) signaling pathway and expression of pro-survival genes (Weinreb et al., 2009), and restoration of reduced PKC and extracellular signal-regulated kinases (ERK1/2) activities caused by 6-OHDA toxicity (Zhao, 2009). Tea and/or EGCG prevent neurotoxin-induced cell injury (Weinreb et al., 2004), MPTP-induced dopaminergic neurodegeneration and restore striatal levels of DA and its metabolites (Levites et al., 2001; Choi et al., 2002). Green tea polyphenols could also protect dopaminergic neurons against MPTP-induced injury by exerting inhibitory effects on DA-transporters, which block the uptake the metabolite MPP+ (Pan et al., 2003a).

In summary, tea consumption seems to be a promising lifestyle choice that may slow age-related deficits and neurodegenerative diseases. Given the evidence from preclinical studies, green tea polyphenols are currently being tested as a treatment for de novo PD patients (ClinicalTrials.gov identifier: NCT00461942).

### Alcohol

Alcohol may exert neuroprotective effects in PD. One case-controlled study found an inverse association between total alcohol consumption and PD (Ragonese et al., 2003). A recent study suggests that low to moderate beer consumption may be associated with a lower PD risk, whereas greater liquor consumption may increase the risk of PD (Liu et al., 2013). Contrary to these findings, most of the epidemiological studies do not support an association between alcohol consumption and risk of PD (Benedetti et al., 2000; Checkoway et al., 2002; Hernan et al., 2003; Palacios et al., 2012b). Currently, the association between alcohol consumption and the risk of PD remains poorly understood.

Despite the conflicting results from epidemiological studies, specific components found in red wine including resveratrol and quercetin, may elicit neuroprotection against PD. Administration of resveratrol or quercetin before MPTP treatment reduced apoptotic cell death and modulated expression of Bax and Bcl-2 in PC12 cultures (Bournival et al., 2009, 2012). Resveratrol has elicited neuroprotective effects by preventing behavioral, biochemical, and histopathological changes that occur in PD animal models (Bureau et al., 2008; Khan et al., 2010). A diet containing resveratrol protects dopaminergic neurons and attenuates motor coordination in MPTP rodent models (Blanchet et al., 2008; Lu et al., 2008). Many studies suggest that the antioxidant actions of resveratrol are responsible for the neuroprotection from MPP+ toxicity (Alvira et al., 2007; Okawara et al., 2007).

Mechanistically, resveratrol reduces inflammation by trapping free radicals and preventing apoptosis of DA-producing neurons (Blanchet et al., 2008; Jin et al., 2008; Lu et al., 2008). *In vitro* studies showed that resveratrol protects DA neurons against LPS-induced neurotoxicity through the inhibition of microglial activation and subsequent pro-inflammatory factors (Zhang et al., 2010). Resveratrol-mediated neuroprotection has also been attributed to the inhibition of nicotinamide adenine dinucleotide phosphate (NADPH) oxidase and possibly activation of SIRT1 (Pallas et al., 2009; Zhang et al., 2010). However, one study suggested that SIRT1 activation does not play a major role in the protective effect of resveratrol against MPP+ cytotoxicity (Alvira et al., 2007). Although the evidence from *in vitro* and animal studies is promising, epidemiological studies do not support an association between red wine consumption and PD (Palacios et al., 2012b). Further research on the type and amount of dietary alcohol intake and the risk of PD would be very beneficial.

## Nutrients with a questionable role in PD

### Fat

Dietary fat has shown inconsistent results in relation to PD. Rodent studies show diets high in fat exacerbate the progression of parkinsonism by exhibiting increased DA depletion in the SN, striatum, and nigrostriatal pathway (Choi et al., 2005b; Morris et al., 2010; Bousquet et al., 2011b). With regards to humans, epidemiological studies found a higher risk of PD among individuals with greater intake of total animal fat (Logroscino et al., 1996; Anderson et al., 1999; Johnson et al., 1999; Chen et al., 2003), whereas other studies show no significant relationship between PD and animal fat (Hellenbrand et al., 1996a; Chen et al., 2002, 2003; Powers et al., 2003). Moreover, the positive association between fat and PD risk reported earlier (Anderson et al., 1999) was not replicated in a larger study (Powers et al., 2003). Nonetheless, the conflicting results from these studies may be attributed to the specific type of fat in the diet, saturated or unsaturated, which is not always specified. Nor is the amount of animal protein consumed to supply the fat intake discussed.

In animal studies and clinical trials, a ketogenic diet, which is high in fat, provided symptomatic and beneficial disease-modifying activity in PD (Gasior et al., 2006). In fact in a small clinical trial, five PD patients on a hyperketonemia diet that substituted unsaturated for saturated fats showed improvement on the Unified Parkinson's Disease Rating Scale (Vanitallie et al., 2005). It should also be noted that the patients on the ketogenic diet ate only 8% protein. Low protein diets lead to better levodopa bioavailability (Pincus and Barry, 1987). It is therefore possible that the observed improvement may have been due to better absorption of synthetic dopamine in four of the patients since one patient was not taking anti-parkinson medication (Vanitallie et al., 2005). Because of the limited number of patients, the difficulty in adhering to a hyperketonemia diet, and the lack of healthy controls, the authors were not able to rule out a placebo effect. The promising results from this preliminary study suggest that another clinical trial of the ketogenic diet that includes a larger number of patients is warranted.

Dietary intake of PUFAs and monounsaturated fatty acids (MUFAs) might influence the risk of PD (Abbott et al., 2003; De Lau et al., 2005). It has been reported in other disease models that PUFA's have anti-inflammatory and neuroprotective properties (Blok et al., 1996; Simopoulos, 1999; Youdim et al., 2000; Kim et al., 2001) and MUFAs are thought to reduce oxidative stress (Colette et al., 2003; Moreno and Mitjavila, 2003). Unsaturated fatty acids are important constituents of neuronal cell membranes and the fatty acid composition of cell membranes is affected by diet. It has been demonstrated in other disease models that infants and young animals with dietary deficiencies in MUFAs and PUFAs have a decrease in brain function (Fernstrom, 1999; Simopoulos, 1999; Youdim et al., 2000; Moreno and Mitjavila, 2003). Moreover, it has been shown that PUFA intake is consistently associated with lower PD risk, and dietary fats modified the association of PD risk with pesticide exposure (Kamel, 2013; Kamel et al., 2013). Notably, PD was inversely associated with the N-3 precursor α-linolenic acid, an essential fatty acid, in a meta-analysis comprising nine studies (Kamel et al., 2013). The health benefit effects of α-linolenic acid may be due to its potential role in protecting against oxidative stress and inflammation (Hassan et al., 2010; Robinson and Mazurak, 2013; Zhang et al., 2013). These studies suggest that a diet high in PUFAs and low in saturated fats might reduce the risk of PD and protect from the toxic effects of neurotoxins, such as those possibly present in milk.

Alternatively, saturated fat could modify the risk of PD by affecting PUFA metabolism and inducing adverse changes in cell membrane lipid composition (Peers, 1997). Thus, fatty acids may contribute to an increased risk of PD via oxidative stress. PUFAs are concentrated in neuronal membranes and play a role in oxidative radical formation. Lipid peroxidation results in oxidative damage and can modify lipid composition of membranes, potentially leading to neuronal death (Farooqui and Horrocks, 1998). In addition, adverse essential fatty acid composition in the mitochondrial membrane may also induce phosphorylation uncoupling, causing energy failure (Peers, 1997). Thus, a high concentration of PUFAs may contribute to neural oxidative stress through lipid peroxidation. Additionally, PD patients have higher concentrations of PUFA peroxidation metabolites and lower concentrations of PUFA and glutathione in the SN compared to healthy controls, further supporting the hypothesis that energy failure may facilitate the onset and/or progression of PD (Chen et al., 2003). However, higher concentrations of PUFA peroxidation metabolites and lower PUFA may arise from several environmental factors in addition to nutrients.

The importance of fats in the pathogenesis of PD in some patients is suggested by genetic studies. Mutations in *PARK2*, which encodes the PD related factor Parkin, lead to an early onset familial form of PD (Kitada et al., 1998). Parkin is part of the E3 ubiquitin ligase complex that targets specific substrates for degradation via the ubiquitin−proteasome pathway (Shimura et al., 2000). Recently, it was shown that Parkin is a lipid-dependent regulator of fat uptake in mice and patient cells carrying mutations in *PARK2* (Kim et al., 2011). These studies suggest that genetic mutations in the uptake or breakdown of fat may be associated with PD.

Lipid and cholesterol metabolism may also play a role in the pathogenesis of idiopathic PD, however the association between cholesterol and PD is highly debated (Hu, 2010). Lower plasma cholesterol concentrations (Lamperti, 1991) and decreased cholesterol biosynthesis is observed in cell lines from PD patients (Musanti et al., 1993), suggesting that low levels of cholesterol may play a role in PD development and/or progression. In contrast, higher total serum cholesterol may be associated with a modest slower progression of PD (Huang et al., 2011) and lower iron content in SN and globus pallidus in PD patients (Du et al., 2012). Interestingly, the association with increased cholesterol levels and decreased PD was seen primarily in women (De Lau et al., 2006). One possible explanation about the lack of an association between cholesterol levels and PD in men may be due to the gender differences of plasma concentration levels of the antioxidant coenzyme Q10 (De Lau et al., 2006), which are significantly higher in men than in women (Kaikkonen et al., 1999). In this regard, it should be noted that coenzyme Q10 has shown neuroprotective properties in numerous PD studies (Shults et al., 2004; Cleren et al., 2008). More recently, the total; HDL cholesterol ratio was found to be inversely associated with disease duration, thereby suggesting an effect of cardiometabolic protection in PD (Cassani et al., 2013). The results from this study must be interpreted with caution since no healthy controls were included in the analysis.

The studies cited above reflect our incomplete understanding regarding the association between fat intake and PD. The role that fat plays in PD is most likely related to the type of fat in the patient's diet (De Lau et al., 2005), the patient's HDL/LDL ratio, total cholesterol levels and genetic factors. Ideally, large prospective randomized controlled studies are needed to clarify the associations between fat intake and PD.

### Meat

Meat is another source of animal fat and its consumption may be associated with the incidence of PD (Anderson et al., 1999) but the evidence from prospective studies is limited (Gaenslen et al., 2008). Interestingly, intake of processed meat and sausages was inversely associated with PD risk in women (Saaksjarvi et al., 2013). This finding is surprising given the higher incidence of mortality, cardiovascular diseases, and diabetes associated with processed meat consumption (Micha et al., 2010; Rohrmann et al., 2013). In the case of red meat, a positive association between red meat consumption and PD may be explained by the heme content that may act as a toxin when not digested properly. Heme is found in other meats also but not to the same extent. Hemin increases intracellular iron concentrations and hydroxyl radical production, contributing to iron deposits and mitochondrial damage (Schipper, 2000). In this context, iron intake from dietary nutrients may be related to risk for PD (Powers et al., 2003) but the evidence for this association is conflictive (Logroscino et al., 1998, 2008). Despite the inconsistent results, higher intake of iron is associated with neuroprotection in PD (Miyake et al., 2011b). Notwithstanding the positive results, the authors of this study noted that evaluation of dietary intake for 1 month prior to completing the questionnaire by the participants might not properly represent their typical diets.

### Carbohydrates

It has been suggested that carbohydrates increase DA production in the brain by allowing easier passage of the DA precursor, tyrosine, through the blood-brain barrier into cerebrospinal fluid (Fernstrom et al., 1979; Wurtman et al., 2003). Carbohydrates with high glycemic index decrease the risk of PD by an insulin-induced increase in brain DA (Murakami et al., 2010a). A balanced diet of carbohydrate and protein mixture improved motor performance in PD patients (Berry et al., 1991). Yet, epidemiological studies about carbohydrate consumption and PD remain inconclusive. For example, the Nurses Health Study and Health Professionals Follow-up Study reported a non-significant direct association in women and inverse association in men for carbohydrate consumption and PD risk (Chen et al., 2003). In contrast, other studies have shown a positive association for total carbohydrate consumption and PD (Hellenbrand et al., 1996a; Abbott et al., 2003).

High carbohydrate diets are associated with an increased risk of type 2 diabetes (T2DM) (Salmeron et al., 1997a,b; Oba et al., 2013). Interestingly, numerous epidemiological studies indicate T2DM is associated with an increased risk of PD (Schernhammer et al., 2011; Xu et al., 2011; Sun et al., 2012; Cereda et al., 2013) but the evidence presented is conflictive (Simon et al., 2007; Palacios et al., 2011; Noyce et al., 2012). Nonetheless, T2DM is associated with more severe motor symptoms in PD (Kotagal et al., 2013). One possible explanation for the link between both chronic diseases is that alterations in common biological pathways may lead to neurodegeneration in patients with T2DM (Santiago and Potashkin, 2013b). In this regard, emerging research is beginning to elucidate the molecular networks and potential mechanisms implicated in both diseases (Santiago and Potashkin, 2013a; Mattson, 2014; Wang et al., 2014). Since carbohydrates are an important part of people's diets and its high consumption may increase risk for T2DM (Salmeron et al., 1997a,b; Oba et al., 2013) further research on the amount and type of dietary carbohydrates consumed in relationship to the risk of PD would be very beneficial.

### Vitamin D, C, and E

Vitamin D deficiency is prevalent in PD patients (Sato et al., 1997); yet, it is unclear if a reduction in Vitamin D is a cause or consequence of PD. Vitamin D plays a role in regulating Ca^2+^ homeostasis (Garcion et al., 2002; Chan et al., 2009) and if disrupted, SNpc dopaminergic neuron loss is accelerated (Gleichmann and Mattson, 2011). This suggests that dietary regulation of vitamin D may be effective in protecting individuals from PD or slowing PD progression. In animal and cell culture models of PD, vitamin D supplementation was found to be beneficial in slowing disease progression (Wang et al., 2001; Smith et al., 2006; Holick, 2007). In human studies, however, high consumption of food containing vitamin D increased the risk of PD (Anderson et al., 1999). More recently, vitamin D3 supplementation stabilized PD patients' motor symptoms, preventing an increase in the Hoehn and Yahr stage, compared to a placebo-controlled group (Suzuki et al., 2013). It remains unknown if a reduction in vitamin D stemming from nutritional deficiencies causes an increase in PD and/or if an environmental factor such as UV radiation or exposure to sunlight plays a role. Therefore, more research needs to be done in order to link vitamin D supplementation and its effective in protecting individuals from PD or PD progression.

Vitamin C or ascorbate is highly concentrated in the central nervous system and its neuroprotective capabilities show promise in reducing lipid peroxidation levels and increasing catalase activity (Santos et al., 2008). Higher intake of vitamin C correlates with an increase risk of PD (Scheider et al., 1997). In contrast, in a case-controlled study, individuals consuming a diet rich in vitamin C showed a 40% reduction of PD risk (Hellenbrand et al., 1996a). Interestingly, in a pilot study in which high doses of vitamin C and E were given to early stage PD patients, a decrease in disease progression was observed (Fahn, 1992). Despite this progress, other studies have not found a significant association between intake of dietary vitamin C or vitamin C supplements and risk of PD (Zhang et al., 2002; Etminan et al., 2005). Collectively, the association of vitamin C and PD risk remains inconclusive and more studies are needed to clarify this association.

Vitamin E supplementation provides protective effects on DA neurons in the SNpc (Roghani and Behzadi, 2001), reduce DA loss (Lan and Jiang, 1997), and protect against paraquat toxicity (Storch et al., 2000; Osakada et al., 2004) in rodents and *in vitro*. Pretreatment with vitamin E reduces lipid peroxidation levels (Lan and Jiang, 1997), but depletion of striatal DA was not attenuated in animals (Gong et al., 1991; Chi et al., 1992). The potential benefits seen in vitamin E may be linked to its chain-breaking capabilities in biological membranes, preventing induced oxidative damage by trapping reactive oxyradicals. Yet, other studies have shown that vitamin E has no protective effects against DA-induced toxicity in PC12 cells (Offen et al., 1996) and only partial protection in MPTP-treated marmosets (Perry et al., 1987). A meta-analysis showed a protective effect against PD in humans with both moderate and high intake of vitamin E (Etminan et al., 2005), with a more significant effect observed in men than women (Zhang et al., 2002). In contrast, clinical trials show no neuroprotective benefits from vitamin E in PD patients (Fernandez-Calle et al., 1992; Lewitt, 1994).

Although researchers have started investigating the effect of individual nutrients through supplements the results of these studies remain inconclusive. Antioxidants are much more effective in combinations and therefore a combination of vitamins may be beneficial, perhaps acting synergistically. Thus, we suggest that choosing a diet that contains a variety of foods that are rich in multiple phytochemicals and other bioconstituents may provide a means of disease management. The total elimination of any one food group is not recommended. Additional prospective nutritional studies should help to resolve this issue.

## Nutrition, the genome, and the epigenome

A poor diet will have a negative impact on an individual's health. With regards to neurodegeneration, nutrition affects multiple aspects of neurodevelopment, neurogenesis and the functions of neurons and neural networks (Dauncey and Bicknell, 1999). Nutrition-gene interactions play a critical role in dysfunction and disease (Dauncey, 2012). Individual differences in genes such as single nucleotide polymorphisms, mutations and copy number variants significantly modify the effects of nutrition on gene expression (Dauncey, 2013).

A person's epigenome is just as important as their genome. An individual's epigenome reflects the interaction of the person's genome with their environment. Epigenomic modifications include DNA methylation, which may alter protein-DNA interaction and result in genes being expressed or turned off. Another type of modification is histone modification, which may lead to changes in DNA packaging. Histone modification may also lead to switching a gene on or off by making the DNA packaging more or less accessible to proteins. In addition, epigenetic regulation of gene expression through small non-coding RNAs is environmentally regulated. Epigenetic regulation of gene expression plays an important role in development and pathological processes (Dauncey et al., 2001; Babenko et al., 2012; Dauncey, 2012; Hackett et al., 2012; Park et al., 2012; Qureshi and Mehler, 2013). What a person eats and drinks will impact their epigenome (Dauncey, 1997, 2012; Langie et al., 2012). Currently the details about how individual nutrients affect the epigenome generally remain unknown. This area of nutrition research is still in its infancy. If we want to improve peoples' health it will be important to emphasize this area of research in the future because epigenetic changes also impact future generations since they may be inherited.

## Conclusions

**Figure 1:**
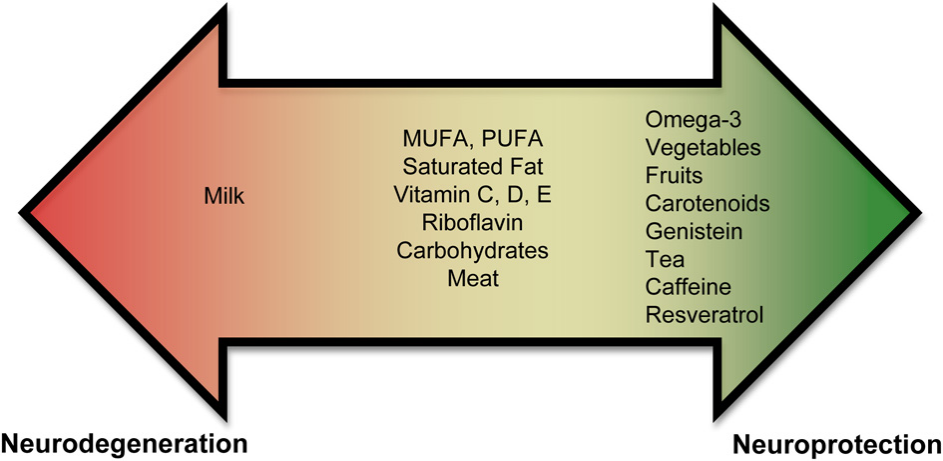
**Role of nutrients in PD**. Epidemiological and biochemical studies suggest that inclusion or exclusion of certain food groups may elicit neuroprotection or neurodegeneration. Foods are shown on a spectrum. Foods shown in the red promote neurodegeneration and foods in green promote neuroprotection. Foods shown in the middle (or yellow) part of the spectrum have conflicting results and need to be studied further to assess if they play a role in neurodegeneration or neuroprotection.

Currently, there is an abundance of preliminary evidence that indicates that some nutrients may increase an individual's risk for PD, while others may be neuroprotective (Figure 1, Supplementary Tables 1, 2). These results are not unexpected since nutrients affect mitochondrial energy function and provide vital antioxidant functions that ameliorate the free-radical byproducts of oxidative phosphorylation. A poor diet may lead to increased oxidative stress, which could impede the antioxidant defense system. In contrast, a well-balanced diet rich in a variety of foods, including numerous servings of vegetables and fruits (especially those containing nicotine) and moderate amounts of omega-3 fatty acids, tea, caffeine, and wine may provide neuroprotection.

In spite of promising effectiveness of these nutrients in PD, we lack definitive evidence-based answers as a result of limited large prospective randomized controlled studies designed to address these issues. Indeed, there are several limitations in some epidemiological studies assessing dietary factors and PD that merit further attention. For example, the assumption that dietary patterns remain unchanged over time is a major limitation. Information on diet during development would be very helpful and may weaken or strength a result. In addition, patients with PD may experience non-motor symptoms at early stages such as constipation, dysphagia, depression, and hyposmia that may affect dietary choices and therefore may be responsible for the impairment of nutritional status observed in PD (Ponsen et al., 2004; Barichella et al., 2009). These factors may remain undetected and therefore not properly reported. Incorporation of these critical factors into clinical practice and epidemiological studies will greatly improve the reliability of studies assessing the role of nutrients in PD.

### Conflict of interest statement

The authors declare that the research was conducted in the absence of any commercial or financial relationships that could be construed as a potential conflict of interest.
