# Supplementary material for: The emerging role of nutrition in Parkinson's disease
Source: Front Aging Neurosci. 2014 Mar 7;6:36. doi: 10.3389/fnagi.2014.00036 (PMC3945400; doi:10.3389/fnagi.2014.00036)
Supplement: Supplementary file 1 [file DataSheet1.PDF]

Supplementary Table 1. Human studies addressing the effects of nutrition in Parkinson's disease.
E represents an epidemiological study and R represents a review.

Classification	Study Results	References
Dairy	**Dairy Constituents**<br>Dairy fat, protein and lactose increases risk of PD.	E (Hellenbrand et al., 1996b;Chen et al., 2002;Park et al., 2005;Chen et al., 2007a;Kyrozis et al., 2013)
	Dairy consumption may increase the risk of PD, particularly in men.	E (Chen et al., 2007a)
	E (Saaksjarvi et al., 2013)	Milk consumption is positively associated with PD risk in women.
	Lack of association between dairy products and risk of PD.	E (Miyake et al., 2011c)
	High consumption of dairy results in low urate levels.	E (Choi et al., 2005a)
	High plasma levels of uric acid may modify the risk of PD.	R (Schlesinger and Schlesinger, 2008)
	**Calcium**<br>Calcium and vitamin D are positively associated with PD risk only when derived from dairy products.	E (Chen et al., 2002)
	**Vitamin D**<br>High consumption of food containing vitamin D increases risk of PD in humans.	E (Anderson et al., 1999)
Fat	**Fat**<br>A higher risk of PD is seen with greater intake of total fat- saturated and animal.	E (Logroscino et al., 1996;Anderson et al., 1999;Chen et al., 2003)
	No association between PD and fat intake was observed.	E (Hellenbrand et al., 1996b;Logroscino et al., 1996;Johnson et al., 1999;Chen et al., 2002;Powers et al., 2003)
	A ketogenic diet provides symptomatic and disease-modifying activity in PD.	R (Gasior et al., 2006)
	A modified ketogenic diet consisting of mono and polyunsaturated fats improved the Unified Parkinson's Disease Rating Scale.	E (Vanitallie et al., 2005)
	Intake of PUFAs is protective for PD as well as MUFAs.	E (Abbott et al., 2003;de Lau et al., 2005)

	**Polyunsaturated fatty acids**<br>PD patients exhibit higher concentrations of PUFA peroxidation metabolites, but lower concentrations of PUFA and glutathione in the SN compared to controls.	E (Chen et al., 2003)
	PUFAs intake is associated with lower PD risk.	E (Kamel et al., 2013)
	**Cholesterol**<br>An association between cholesterol and PD is highly debated.	R (Hu, 2010)
	Lower plasma cholesterol concentrations and biosynthesis is found in PD patients compared to controls and that statins use may lower PD ocurrence.	E (Lamperti, 1991)
	Increased cholesterol levels are associated with lower PD risk, primarily in women.	E (de Lau et al., 2006)
	Higher total serum cholesterol may be associated with a modest slower progression of PD.	E (Huang et al., 2011)
	Total HDL-cholesterol ratio is inversely associated with duration of PD and may contribute to cardiometabolic protection.	E (Cassani et al., 2013)
Fruits and Vegetables	Nicotine-containing vegetables from edible Solanaceae are associated with a reduced risk of PD.	E (Searles Nielsen et al., 2013)
	A dietary pattern including high consumption of fruits, vegetables and fish are inversely associated with PD risk.	E (Gao et al., 2007;Okubo et al., 2012)
	No relationship was found between intake of vegetables or fruit and the risk of PD.	E (Miyake et al., 2011a)
	**Vitamin C**<br>Higher intake of fruits and certain vegetables containing vitamin C is associated with an increased risk of PD.	E (Scheider et al., 1997)
	No significant association between vitamin C from food and PD.	E (Zhang et al., 2002;Etminan et al., 2005)
	**Vitamin E**<br>Vitamin E from food is associated with a lower risk of PD, particularly in men.	E (Zhang et al., 2002)

Carbohydrates	High glycemic index foods decrease the risk of PD.<br><br>Carbohydrate consumption and PD risk is reported with a non-significant direct association in women and inverse association in men.<br><br>Total carbohydrate consumption is positively associated with PD.	E (Murakami et al., 2010a)<br><br>E (Chen et al., 2003)<br><br>E (Hellenbrand et al., 1996a)
Protein	**Protein**<br>No correlation is found with total protein intake and the risk of PD.<br><br>Elimination of dietary red meat accompanied with high doses of riboflavin promotes recovery of motor functions in PD patients.	E (Hellenbrand et al., 1996b;Logroscino et al., 1996;Johnson et al., 1999;Chen et al., 2003)<br><br>E (Coimbra and Junqueira, 2003)
Beverages	**Caffeine**<br>Caffeinated beverages may provide neuroprotection against PD.<br><br>Coffee drinking in women who are not taking hormone-replacement therapy is associated with a reduced risk in PD as observed in men.<br><br>Clinical studies are underway to evaluate several A_{2A} receptor antagonists for symptomatic relief and slowing of disease progression in PD.<br><br>Caffeine appears to improve motor and non-motor conditions in PD clinical trials. Gastrointestinal discomfort and anxiety were common adverse effects.<br><br>**Tea**<br>Black tea drinking shows an inverse association with PD risk whereas green tea was unrelated to PD risk in a Chinese population.<br><br>Tea consumption may reduce the risk of PD.<br><br>Tea consumption delayed onset of motor symptoms in PD patients.<br><br>**Alcohol**<br>No association between alcohol consumption and PD.	R (Prakash and Tan, 2011)<br><br>E (Ascherio and Chen, 2003;Palacios et al., 2012a)<br><br>R (Barkhoudarian and Schwarzschild, 2011;Hickey and Stacy, 2011)<br><br>E (Altman et al., 2011)<br><br>E (Tan et al., 2008)<br><br>E (Chan et al., 1998;Checkoway et al., 2002)<br><br>E (Kandinov et al., 2009)<br><br>E (Benedetti et al., 2000;Checkoway et al., 2002;Hernan et al., 2003;Palacios et al., 2012b)

	Inverse association between alcohol consumption and PD.<br><br>Low to moderate beer consumption may lower the risk of PD whereas high liquor consumption may increase PD risk.	E (Ragonese et al., 2003)<br><br>E (Liu et al., 2013)
Supplements	**Beta-carotenoids**<br>Higher intake of beta-carotenoids is associated with a decreased risk of PD in women.<br><br>**Vitamin B - Riboflavin**<br>Daily doses of riboflavin for 6 months show improved motor capacity in PD patients in 3 months.<br><br>Riboflavin is not associated with risk of PD.<br><br>Low intake of vitamin B6 is associated with an increased risk of PD.<br><br>Folate, vitamin B6 and B12 are not associated with a risk of PD.<br><br>**Vitamin C**<br>Vitamin C is not associated with PD risk.<br><br>**Vitamin D**<br>Intake of vitamin D is not associated with PD risk.<br><br>Vitamin D3 supplementation prevented the deterioriation of the Hoehn & Yahr stage in PD patients compared to placebo-controlled group.<br>**Vitamin E**<br>Vitamin E is not associated with PD risk.<br><br>Clinical trials show no neuroprotective benefit of taking vitamin E.<br><br>Higher intake of vitamin E is associated with a reduced risk of PD in women.	E (Miyake et al., 2011a)<br><br>E (Coimbra and Junqueira, 2003)<br><br>E (Abbott et al., 2003;Murakami et al., 2010b)<br><br>E (Murakami et al., 2010b)<br><br>E (Chen et al., 2004)<br><br>E (Zhang et al., 2002) E (Miyake et al., 2011a)<br><br>E (Chen et al., 2002)<br><br>E (Suzuki et al., 2013)<br><br>E (Zhang et al., 2002)<br><br>E (Fernandez-Calle et al., 1992;LeWitt, 1994)<br><br>E (Miyake et al., 2011a)

Supplementary Table 2. Animal and *in vitro* studies addressing the effects of nutrients in Parkinson's disease. A is animal and IV is *in vitro*.

Classification	Study Result	Reference
Fat	A high fat diet exacerbates the progression of PD in rodents by increasing DA depletion and damage.<br><br>Polyunsaturated fatty acids inhibit neuronal apoptosis in cellular models.	A (Choi et al., 2005b;Morris et al., 2010;Bousquet et al., 2011b)<br><br>IV (Kim et al., 2001)
Docosahexaeonic acid (DHA)	DHA reduces apoptosis in dopaminergic cells and preserves DA levels from MPTP-induced neurotoxicity in mice.<br><br>DHA protected neurons against cytotoxicity, inhibition of NO production, Ca^{2+} influx, and increased the activities of antioxidant enzymes glutathione peroxidase and glutathione reductase.<br><br>Short-term administration of DHA reduced levodopa-induced dyskinesias in Parkinsonian primates.<br><br>DHA treatment elevated DA levels in a 6-OHDA model of PD.<br><br>DHA supplementation replaces omega-6-PUFAs already present in the brains post-MPTP treatment.	IV (Ozsoy et al., 2011)<br>A (Bousquet et al., 2008)<br><br>A (Wang et al., 2003)<br><br>A (Samadi et al., 2006)<br><br>A (Cansev et al., 2008)<br><br>A (Bousquet et al., 2008)
Eicosapentaenoic acid (EPA)	EPA attenuated motor impairments, and inflammation in a MPTP model of PD.	A (Luchtman et al., 2012)<br>IV (Luchtman et al., 2013)
Caffeine	Chronic caffeine administration in mice provided protection against dopaminergic neuron toxicity from exposure to a combination of common pesticides- Paraquat and Maneb.<br><br>Acute and chronic caffeine administration reduced the effect of acute MPTP and 6-OHDA treatment on striatal DA loss.<br><br>Caffeine treatment partially restored DA and its metabolites in 6-OHDA-lesioned rats.<br><br>Caffeine is neuroprotective in MPTP model of PD.	A (Kachroo et al., 2010;Yadav et al., 2012)<br><br>A (Chen et al., 2001;Joghataie et al., 2004)<br><br>A (Aguiar et al., 2006)<br><br>A (Xu et al., 2010)

	Chronic caffeine treatment prevented DA cell degeneration in a MPTP model of PD. Neuroprotection was still observed after the onset of PD.<br><br>Caffeine reduces neurotoxicity through antagonism of adenosine A_{2A} receptors.<br><br>Methylxanthine A_{2A} receptor antagonists may cause oxidative stress in PD.	A (Sonsalla et al., 2012)<br><br>R (Morelli et al., 2010;Prediger, 2010)<br><br>A (Golembiowska and Dziubina, 2012)
Soy	Pre-treatment of parkinsonian rats with dietary soy meal improved spatial learning and memory.<br><br>Genistein appears to be neuroprotective in ovariectomized rats, thereby suggesting it may be useful for prevention of PD in post-menopausal women.<br><br>Genistein protects dopaminergic neurons from lipopolysaccharide-induced injury.<br><br>Pre-treatment with genisten restored MPTP-induced down regulation of TH, dopamine transporter and Bcl-2 mRNA expression in the midbrain.<br><br>Genistein administration attenuated the rotational behavior in lesioned rats and protected neurons against 6-OHDA toxicity.	A (Sarkaki et al., 2009)<br><br>A (Kyuhou, 2008)<br><br>IV (Wang et al., 2005)<br><br>A (Liu et al., 2008)<br><br>A (Baluchnejadmojarad et al., 2009)
Polyphenols	**EGCG**<br>The green tea polyphenol EGCG is neuroprotective by preventing neurotoxin-induced cell injury and prevent MPTP-induced dopaminergic neurodegeneration.<br><br>EGCG provides neuroprotection through nitric oxide reduction.<br><br>Oral treatment with EGCG provided symptomatic relief but no neuroprotection in 6-OHDA model of PD.<br><br>**Quercetin**<br>Quercetin prevents apoptosis of DA-producing neurons.<br><br>**Resveratrol**<br>Resveratrol reduces deterioration caused by free radicals preventing subsequent behavioral, biochemical, and histopathological changes that	R (Mandel et al., 2004)<br>A (Levites et al., 2001)<br><br>A (Kim et al., 2010)<br><br>A (Leaver et al., 2009)<br><br>IV (Bureau et al., 2008)<br><br>IV (Bureau et al., 2008)

	occur during PD.<br><br>Resveratrol prevents apoptosis of DA-producing neurons and exerts neuroprotective effects on 6-OHDA-induced animals by reducing inflammatory reactions as well scavenging free radicals in MPTP.<br><br>Theaflavin mediated neuroprotection in MPTP model of PD.<br><br>**Carotenoids**<br>Pretreatment with beta-carotene partially protected against MPTP-induced neurotoxicity in mice, but not in primates.<br><br>Lycopene reduces oxidative stress and cognitive decline in a rotenone induced model of PD.<br><br>**Sulforaphane**<br>Ameliorated motor deficits prevented dopaminergic cell death by modulating oxidative stress.<br><br>**Erucin**<br>Treatment with erucin provided neuroprotective effects against 6-OHDA in a neuronal cell culture model.	 <br>A (Blanchet et al., 2008;Jin et al., 2008;Lu et al., 2008)<br><br>A (Anandhan et al., 2012)<br><br>A (Perry et al., 1985;Perry et al., 1987)<br><br>A (Kaur et al., 2011)<br><br>A (Morroni et al., 2013)<br><br>IV (Tarozzi et al., 2012)
Wheat germ	Wheat germ oil is neuroprotective in 6-OHDA model of PD.	A (Wang et al., 2010)
Vitamin D	Vitamin D is beneficial in animal and cell culture models of PD.	A (Wang et al., 2001;Smith et al., 2006)<br>IV (Holick, 2007)
Vitamin E	Vitamin E supplementation protected DA neurons in the SNpc, reduced DA loss and showed protection against paraquat toxicity.	A (Lan and Jiang, 1997;Storch et al., 2000a;Roghani and Behzadi, 2001)
Curcumin and naringen	Curcumin and naringenin promote neuroprotection in PD.	IV (Chen et al., 2006)<br>A (Zbarsky et al., 2005;Rajeswari, 2006)

Supplementary Table 2. Animal and *in vitro* studies addressing the effects of nutrients in Parkinson's disease. A is animal and IV is *in vitro*.

References

Ascherio, A., and Chen, H. (2003). Caffeinated clues from epidemiology of Parkinson's disease. Neurology 61, S51-54.

Chen, J., Tang, X.Q., Zhi, J.L., Cui, Y., Yu, H.M., Tang, E.H., Sun, S.N., Feng, J.Q., and Chen, P.X. (2006). Curcumin protects PC12 cells against 1-methyl-4-phenylpyridinium ion-induced apoptosis by bcl-2-mitochondria-ROS-iNOS pathway. Apoptosis : an international journal on programmed cell death 11, 943-953.

Mandel, S., Weinreb, O., Amit, T., and Youdim, M.B. (2004). Cell signaling pathways in the neuroprotective actions of the green tea polyphenol (-)-epigallocatechin-3-gallate: implications for neurodegenerative diseases. Journal of neurochemistry 88, 1555-1569.

Rajeswari, A. (2006). Curcumin protects mouse brain from oxidative stress caused by 1-methyl-4-phenyl-1,2,3,6-tetrahydropyridine. European review for medical and pharmacological sciences 10, 157-161.

Storch, A., Kaftan, A., Burkhardt, K., and Schwarz, J. (2000a). 1-Methyl-6,7-dihydroxy-1,2,3,4-tetrahydroisoquinoline (salsolinol) is toxic to dopaminergic neuroblastoma SH-SY5Y cells via impairment of cellular energy metabolism. Brain research 855, 67-75.

Wang, T., Liu, Y.Y., Wang, X., Yang, N., Zhu, H.B., and Zuo, P.P. (2010). Protective effects of octacosanol on 6-hydroxydopamine-induced Parkinsonism in rats via regulation of ProNGF and NGF signaling. Acta pharmacologica Sinica 31, 765-774.

Zbarsky, V., Datla, K.P., Parkar, S., Rai, D.K., Aruoma, O.I., and Dexter, D.T. (2005). Neuroprotective properties of the natural phenolic antioxidants curcumin and naringenin but not quercetin and fisetin in a 6-OHDA model of Parkinson's disease. Free radical research 39, 1119-1125.
